# Identification of Transcription Factors Regulating Detoxification Genes *CYP9Z140*, *CYP9AY1*, and *UGT321AP1* Involved in Thiamethoxam Resistance in *Leptinotarsa decemlineata*

**DOI:** 10.3390/insects17050525

**Published:** 2026-05-20

**Authors:** Zhen Tian, Li Liu, Qiuping Zhang, Dongdi Zhou, Kaiyun Fu, Zunzun Jia, Weihua Jiang

**Affiliations:** 1 State Key Laboratory of Agricultural and Forestry Biosecurity, College of Plant Protection, Nanjing Agricultural University, Nanjing 210095, China; m15937095064@163.com (Z.T.);; 2 Key Laboratory of Integrated Pest Management on Crops in Northwestern Oasis, Ministry of Agriculture/Xinjiang Key Laboratory of Agricultural Biosafety, Institute of Plant Protection, Xinjiang Academy of Agricultural Sciences, Urumqi 830091, China

**Keywords:** *Leptinotarsa decemlineata*, thiamethoxam, resistance, transcription factor, cytochrome P450, UDP-glycosyltransferase

## Abstract

The Colorado potato beetle, *Leptinotarsa decemlineata*, is a major insect pest of potatoes. Our previous studies have demonstrated that two cytochrome P450 monooxygenase (P450s) genes, *CYP9Z140* and *CYP9AY1*, and a uridine diphosphate–glycosyltransferase (UGT) gene, *UGT321AP1*, play important roles in thiamethoxam resistance to *L*. *decemlineata*. However, the related upstream regulatory mechanism remains unclear. In this study, we first monitored the resistance of *L*. *decemlineata* field populations to thiamethoxam in Xinjiang to determine the resistance ratios. The predicted results demonstrated that four transcription factors (TFs), CncC/Maf, Abd-B, FoxO, and Ptx1, may bind to the core regions of three gene promoters. The qRT-PCR results revealed that the TFs were significantly upregulated by thiamethoxam and exhibited specific spatiotemporal expression patterns. Dual-luciferase reporter assays indicated that the CncC pathway could regulate the expression of three detoxification genes, whereas Abd-B and FoxO only regulate *CYP9Z140* and *UGT321AP1* expressions, respectively. Ptx1 could regulate the expression of both *CYP9AY1* and *UGT321AP1*. Furthermore, knockdown of several TFs through RNA interference significantly reduced expression of the corresponding detoxification genes, consistent with the dual-luciferase reporter assay results, and increased the thiamethoxam susceptibility of test adults. These findings aid in gaining a deeper understanding of the transcriptional regulation mechanisms of insecticide resistance in insects.

## Simple Summary

*Leptinotarsa decemlineata* is a major agricultural pest of potato that has developed resistance to many insecticides. We previously confirmed that detoxification genes *CYP9Z140*, *CYP9AY1*, and *UGT321AP1* are involved in resistance to the neonicotinoid insecticide thiamethoxam in *L. decemlineata*. In this study, by investigating the promoters of three genes using dual-luciferase reporter assays, we identified binding sites for several xenobiotic transcription factors (TFs), CncC/Maf, Abd-B, FoxO, and Ptx1. The knockdown of the candidate TFs via RNA interference led to downregulation of corresponding detoxification genes and an increase in thiamethoxam susceptibility. In addition, the expression of the candidate TFs was significantly enhanced after exposure to thiamethoxam. Overall, our findings demonstrated that CncC/Maf regulates all three genes, whereas Abd-B, FoxO, and Ptx1 regulate specific subsets to detoxify thiamethoxam in *L. decemlineata*, thus deepening our understanding of the insecticide resistance mechanisms of *L. decemlineata*.

## 1. Introduction

The Colorado potato beetle (CPB), *Leptinotarsa decemlineata* Say (Coleoptera: Chrysomelidae), is a major pest insect of potato crops, causing complete defoliation and resulting in yield losses of up to 60% in the absence of control measures. With the intensive use of insecticides, particularly the neonicotinoids imidacloprid and thiamethoxam, the incidence of related resistance in *L*. *decemlineata* has increased [1]. The CPB has shown resistance to 58 active ingredients (a.i.) from different chemical groups in 144 agricultural regions (pesticide resistance.org). In light of these challenges, it is therefore necessary to determine the level of resistance to the various insecticides currently in use. In China, the beetle is mainly distributed in potato-growing areas north of the Tianshan Mountains in Xinjiang. Through investigations conducted in recent years, we found that the majority of CPB field populations in Xinjiang had developed moderate resistance to thiamethoxam, the main insecticide used locally to control the beetle [2,3].

The results of previous studies have revealed that overexpressed genes of detoxification enzymes, such as cytochrome P450, glutathione S-transferase (GST), uridine diphosphate–glycosyltransferases (UGTs), and ABC transporter proteins, are involved in the resistance of the beetle to imidacloprid. For example, the imidacloprid-resistant CPB constitutively overexpresses multiple P450 genes, including *CYP4Q3*, CY*P9Z26*, *CYP9Z29*, and *CYP6K1*, some of which have been confirmed to be involved in resistance to imidacloprid [4,5,6,7,8,9]. Moreover, a UTG gene, *UGT2*, and several ABC transporter genes, *ABCH278B*, *ABCH278C*, and *ABCG1041A*, have also been found to be overexpressed at higher levels in imidacloprid-resistant CPB, with RNAi-mediated knockdown of the genes found to increase the susceptibility of the beetle to imidacloprid [7,9]. In subsequent studies, researchers identified higher expression of transcription factor Cap ‘n’ collar isoform C (CncC) in the imidacloprid-resistant CPB compared to the susceptible CPB, with knockdown of CncC resulting in downregulation of genes coding for P450 enzymes and ABC transporters and enhancing imidacloprid susceptibility, indicating that CncC is one of the major regulators of the genes responsible for imidacloprid resistance in CPB [8,10]. Similarly, we previously reported that the overexpression of a UGT gene, *UGT321AP1*, in addition to P450 genes, *CYP9Z140* and *CYP9AY1*, contributed to the development of thiamethoxam resistance in *L. decemlineata* [3]. However, the underlying mechanism of transcriptional regulation has yet to be clarified.

Transcription factors primarily include three superfamilies, namely, nuclear receptor (NR), basic helix-loop-helix-PER-ARNT-SIM domain (bHLH-PAS), and basic leucine zipper (bZIP) [11]. In a number of studies, researchers have identified Cap ‘n’ Collar isoform-C/Muscle aponeurosis fibromatosis (CncC/Maf), belonging to the bZIP family, as an important transcription factor for regulating the expression of detoxification genes that are induced in response to xenobiotic compound treatment [11,12,13]. Moreover, ecdysone receptors (EcRs), as important NR members, can regulate the expression of *GSTe1* and *GSTe16*, which are key genes involved in the metabolism of indoxacarb and chlorpyrifos in *Spodoptera litura* [14]. The bHLH-PAS protein family member AhR/ARNT pathway can regulate the expression of *TcFE4* and *TcUGT2B7* to develop resistance to imidacloprid and deltamethrin in *Tribolium castaneum* [15].

Numerous transcription factors and kinases are involved in energy regulation, among which forkhead box O (FoxO) and AMP-activated protein kinase (AMPK) are the main modulators. As the main downstream effector of the insulin receptor substrate/phosphoinositide 3-kinase/protein kinase B (IRS/PI3K/PKB) pathway, FoxO is a highly conserved transcription factor and plays important roles in cellular homeostasis [16,17]. Additionally, the FoxO signaling pathway has been extensively studied in the regulation of detoxification enzyme gene expression and insecticide sensitivity. For example, TcAMPK-TcFOXO signaling positively regulated the transcription of *Tc4EBP* involved in the development of *T. castaneum* in response to deltamethrin treatment [18]. TcFOXO was confirmed as the transcription factor that regulates the expression of *TcCYP6BQ6*, *TcCYP6BQ9*, and *TcCYP6BQ12*, which are involved in the deltamethrin insensitivity of *T. castaneum* [19]. FoxO was not differentially expressed in the susceptible strain or two resistant strains; however, it mediated nitenpyram resistance in *Nilaparvata lugens* through the differential regulation of *CYP4CE1* expression in these three strains [20].

Moreover, gene expression may be regulated by other types of TFs [11,21]. Abdominal-B (Abd-B) belongs to the *Bithorax complex* (BX-C) gene cluster of the Hox gene family, which mainly determines the morphological characteristics and appendage development of the insect posterior segment [22]. The homeodomain transcription factor Ptx1 pituitary homeobox gene (*Ptx1*) is a member of a subgroup of Hox genes and belongs to the *paired*-type homeobox family. Ptx1 has been identified as a key regulator of numerous developmental processes in mammals and *Drosophila*. *D-Ptx1* of *Drosophila* was characterized by a lysine residue at position 9 of the third a-helix of the homeodomain and is expressed at various restricted locations throughout embryogenesis [23]. *Drosophila Ptx1* RNAi counteracts the age-associated downregulation of protease expression, improves muscle protein quality control in a protease-dependent manner, and extends lifespan, implying that Ptx1 plays an important role in maintaining proteostasis during aging [24]. Other research groups have found that B-H_2_ and Ubx, which belong to the Hox gene family of transcription factors, influenced the insecticide sensitivity of insects by regulating the expression of P450 genes [25,26]. However, the exact relationship between Abd-B and Ptx1 and insecticide resistance remains unclear.

In this study, we focused on exploring the upstream regulatory pathways of *CYP9Z140*, *CYP9AY1*, and *UGT321AP1* expressions. The prediction revealed that the core sequences of the promoters of three detoxification enzyme genes have binding sites for the four transcription factors, CncC/Maf, Abd-B, Foxo, and Ptx1. Though analyzing expression profiles and validation experiment results, CncC/Maf was identified as an important transcription factor of the three resistance-related genes. Moreover, Abd-B, Foxo, and Ptx1 could also specifically regulate the expression of the three detoxification genes, respectively. These findings are of significance for more comprehensively understanding the molecular mechanisms of *L*. *decemlineata* in adapting to insecticide.

## 2. Materials and Methods

### 2.1. Insects

Three CPB populations were collected from different potato fields in Xinyuan County (XY24) and Jimusa County (JMSS, JMSZ24) in Xinjiang from June to August 2024 (Table 1). The formulation Alikat, which contains the active ingredient thiamethoxam, is the main insecticide used in these collection areas for controlling the beetles. The URMQY population was collected from Urumqi City in Xinjiang in June 2021 and regarded as a susceptible population without insecticide application (Table 1). The CPBs were subsequently transferred to the lab and reared under the following conditions: 26 ± 1°C, 50–60% relative humidity, and a 16 h/8 h light/dark cycle. They were fed with leaves of the potato variety Xisen No. 6 provided by the Integrated Testing Farm of the Xinjiang Academy of Agricultural Sciences. Male and female adults (3–7 days after emergence) were selected for subsequent analysis.

### 2.2. Bioassay

The contact toxicity of thiamethoxam to CPB adults was determined using a topical application method. Firstly, thiamethoxam (97% powder, Jiangsu Bangsheng Biotechnology Co., Ltd., Huaian, Jiangsu province, China) was diluted to at least five serial concentrations with analytical-grade acetone to induce a 0–100% mortality among the test insects. Next, 1.1 µL of insecticide solution was applied to the ventral area of each adult with a microapplicator (Hamilton Company, Reno, NV, USA). The same volume of acetone treatment was used as a control. The ten treated adults were placed in Petri dishes (9 cm in diameter and 1.5 cm in height) containing fresh potato leaves under the aforementioned conditions. Three replicates were performed for each treatment. The standard reference for dead beetles was based on the method of Liu et al. (2011) [27], and mortality was recorded after 72 h.

### 2.3. Preparation of Samples for Expression Analysis

Three adults were sampled from the survivors of the XY24 population treated with either the LD_50_ of thiamethoxam or acetone (as a control) for 72 h to determine the effect of stress on the expression of the candidate transcription factor genes. To compare the stage-specific expression of the candidate genes, we collected 30 eggs (E), 30 first-instar larvae (L1), 20 second-instar larvae (L2), 10 third-instar larvae (L3), 3 fourth-instar larvae (L4), 3 pupae (P), and 3 adults (A) from the XY24 population. To determine the tissue expression profiles of the candidate genes, the foreguts (FGs), midguts (MGs), hindguts (HGs), Malpighian tubules (MTs), and fat bodies (FTs) were dissected from five adults of XY24, respectively. For each of the above treatments, three biological replicates were performed. All samples were frozen rapidly in liquid nitrogen and stored at −80 °C until use.

### 2.4. Total RNA Isolation and cDNA Synthesis

Total RNA was isolated using Yfx Total RNA Extraction Reagent (Yi Fei Xue Biotechnology Co., Ltd., Nanjing, China), following the manufacturer’s protocol. The concentration of the RNA samples was analyzed on a NanoDrop 1000 spectrophotometer (Thermo Fisher Scientific, Waltham, MA, USA), and its integrity was confirmed using 1.5% agarose gel electrophoresis. The first-strand cDNA was then synthesized by using a PrimeScript RT reagent kit (TaKaRa Biotechnology Co., Ltd., Dalian, China).

### 2.5. Cloning and Bioinformatics Analysis of Candidate Transcription Factors

The open reading frames of Abd-B, FoxO, and Ptx1 were cloned and verified by means of reverse transcription PCR (RT-PCR) using the cDNAs produced from total RNAs of *L*. *decemlineata*, with the primers designed according to the sequences (GenBank accession number: XM_023164461.1, XM_023161366.1, and XM_023163918.2, respectively) listed in Table A2. Multiple alignments of sequences were performed using GeneDoc v2.7.0. The structural domains were detected based on comparison with other identified sequences using the NCBI CD-search online program (https://www.ncbi.nlm.nih.gov/Structure/cdd/wrpsb.cgi, accessed on 30 March 2025). The phylogenetic trees of the three TFs were generated via the neighbor-joining method (1000 bootstrap replicates) in MEGA 7 software based on the amino acid sequences of the three TFs from other insects acquired through similarity searches of the NCBI database.

### 2.6. Real-Time Quantitative PCR

The transcript levels of the candidate transcription factor genes were determined using a Biosystems 7500 Real-time PCR System (Applied Biosystems Inc., Foster City, CA, USA) with ChamQTM SYBR qPCR Master Mix (Vazyme Biotech Co., Ltd., Nanjing, Jiangsu, China). QPCR mixtures comprised 10 µL SYBR Green, 0.4 µL of each primer (10 µmol/L), 1 µL cDNA template (300 ng/µL), and 8.2 µL RNase-free water. The reaction involved the following steps: an initial step at 95 °C for 30 s, followed by 40 cycles of 95 °C for 5 s and 60 °C for 34 s. The primers used are detailed in Table 2. Three independent biological replicates were performed for each qPCR experiment. The relative expression of the target genes was calculated based on the 2^−∆∆CT^ method according to Livak & Schmittgen 2001 [28], with ribosomal protein L4 (*RPL4*) and translation elongation factor 1α (*EF1α*) used as reference genes (average expression stability value 0.326), based on the method of Zhu et al. [29].

### 2.7. RNA Interference

The double-stranded (ds)RNAs of all of the candidate transcription factor genes, including *Abd-B*, *FoxO*, *Ptx1*, *CncC*, *Maf*, and *Keap*, were expressed using Escherichia coli HT115 (DE3) competent cells lacking RNase III, following the methods of Wang et al. (2024) [3], and then diluted to a final concentration of 0.5 μg/μL. dsRNA was quantified using a NanoDrop 1000 spectrophotometer. Potato leaves of similar size were dipped in bacterial solutions for 30 min and then placed in plastic feeding chambers of 17 cm in length, 11.7 cm in width, and 5 cm in height until subjected to air drying. Adults from the XY24 population were carefully transferred into each chamber. To silence the *CncC*, *Maf*, and *Keap* genes simultaneously, the beetles were fed a mixture of ds*RNA* from the three genes at a ratio of 1:1 or 1:1:1 (*v*/*v*). Insects fed on ds*GFP* were used as controls. The experiments were repeated independently six times, and each replicate included 30 insects. A fresh supply of treated potato leaves was provided daily. After 6 d of continuous feeding on treated leaves, RT-qPCR assays were performed to determine the transcription levels of the candidate TFs and *CYP9Z140*, *CYP9AY1*, and *UGT321AP1*. The remaining beetles from each treatment group were treated with a median lethal dose (LD_50_) of thiamethoxam (0.2647 µg/adult), with the same amount of acetone used as a control to determine thiamethoxam susceptibility. Each treatment was repeated four times with 15–20 adults each. The number of dead beetles in each group was then recorded, as described in Section 2.2.

### 2.8. Luciferase Transient Expression Assays

Genomic DNA was extracted from three adults of the XY24 field population of *L*. *decemlineata* using the TIANamp Genomic DNA Kit (TIANGEN Biotech Co., Ltd., Beijing, China) following the manufacturer’s protocol. The first nucleotide (A) of the initiation codon (ATG) of each gene was indicated by ‘+1’, with the upstream region denoted by ‘–’ and the downstream region denoted by ‘+’. The promoters of *CYP9Z140*, *CYP9AY1*, and *UGT321AP1* were amplified and verified by means of RT-PCR with specific primer pairs (Table A1) based on the corresponding promoter sequences obtained from the *L. decemlineata* genome (https://www.ncbi.nlm.nih.gov/assembly/1424361, accessed on 30 May 2024). Subsequently, the promoter fragments of different lengths from the three genes were amplified and cloned into the pGL4.10-basic plasmid containing the firefly luciferase gene using the ClonExpress II One Step Cloning Kit (Vazyme Biotechnology Co., Ltd., Nanjing, China), with open reading frames (ORFs) of predicted TFs, including Abd-B, FoxO, Ptx1, and the CncC pathway, inserted into the pIEx/Bac-4 (pIEx-4) plasmid. All sequences of the cloned promoter fragments were sent to General Biology Co., Ltd. (Chuzhou, Anhui, China) for confirmation. Plasmids were extracted using EndoFree Mini Plasmid Kit II (TIANGEN Biotech Co., Ltd., Beijing, China).

Twelve hours before transfection, approximately 6 × 10^5^ S2 cells per well were added to a 24-well plate and allowed to attach overnight at 27 °C. Empty pGL4.10-basic and pGL4.10-basic-promoter constructs were transfected separately or co-transfected with the pIEx-4-TF plasmid into S2 cells, using Lipofectamine™ 3000 (Thermo Fisher Scientific, Waltham, MA, USA). The pGL4.10-basic plasmid was used as the control. Cells were collected 48 h after plasmid transfection. Relative luciferase activity was assayed using the Dual-Luciferase Reporter Assay kit (Vazyme Biotechnology Co., Ltd., Nanjing, China) and SYNERGY H1 Microplate Reader (BioTek Instruments, Inc., Winooski, VT, USA) and normalized to the level of Renilla luciferase activity. All dual fluorescein reporter experiments were independently repeated at least three times.

### 2.9. Statistical Analysis

Median lethal doses (LD_50_) and 95% fiducial limits (FLs) were determined using PoloPlus 2.00 software (Leora Software, Berkeley, CA, USA). Luciferase activities, mortality, and gene expression levels are expressed as the mean ± standard error (SE) from at least three biological replicates. Student’s *t*-test and one-way analysis of variance (ANOVA) followed by Tukey’s multiple comparison tests were used to analyze the difference between two groups and multiple groups of normally distributed data, respectively. Statistical analysis was performed using GraphPad Prism 8.02 and SPSS Statistics (IBM SPSS Statistics 27 software, Chicago, IL, USA).

## 3. Results

### 3.1. Susceptibility of Different Beetle Populations to Thiamethoxam

The assay results of different field populations of the beetle from Xinjiang in 2024 are shown in Table 3. Compared with the susceptible population, URMQY, our findings showed that JMSS24 and XY24 exhibited low levels of resistance to thiamethoxam, with resistance ratios (RRs) of 6.69- and 8.51-fold, respectively, whereas JMSZ24 remained susceptible.

### 3.2. Analysis of the Key Regions of Three Gene Promoters and TF Prediction

**Figure 1:**
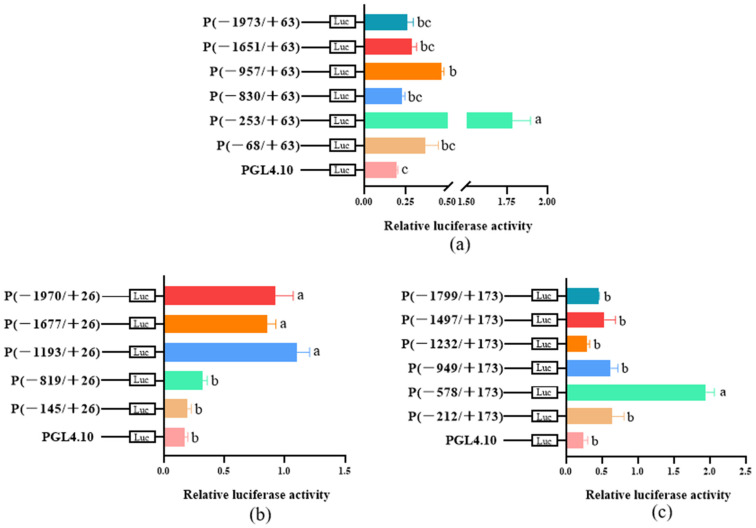
Activity analysis of serial deletion fragments of the 5′ regulation region in (**a**) *CYP9Z140*, (**b**) *CYP9AY1*, and (**c**) *UGT321AP1* of *L. decemlineata.* The pGL4.10-basic plasmid was used as the control. The relative luciferase activity is presented as mean ± SE of three independent transfections. Bars with different lowercase letters indicate significant differences based on one-way ANOVA followed by Tukey’s multiple comparison test (*p* < 0.05).

**Figure 2:**
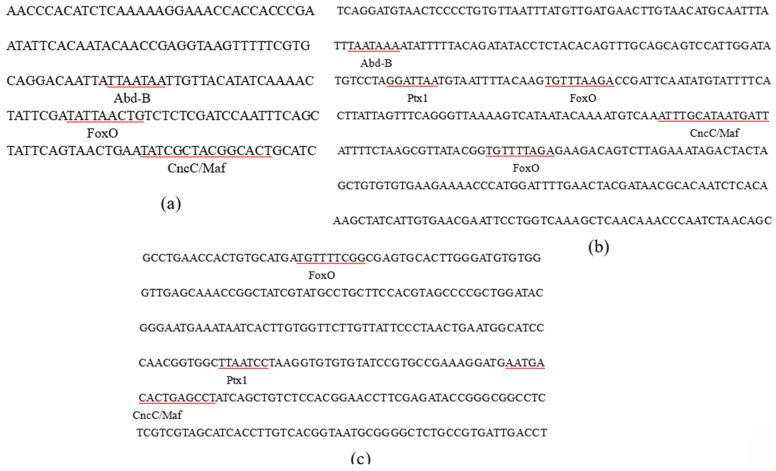
Core promoter regions of three detoxification enzyme genes and the predicted transcription factor-binding sites in *L. decemlineata*. (**a**) Predicted transcription factor-binding sites in the −253 to −68 bp region of the 5′ flanking sequence of *CYP9Z140*; (**b**) Predicted transcription factor-binding sites in the −1193 to −819 bp region of the 5′ flanking sequence of *CYP9AY1*; (**c**) Predicted transcription factor-binding sites in the −578 to −212 bp region of the 5′ flanking sequence of *UGT321AP1*.

The results of our previous study demonstrated that *CYP9Z140*, *CYP9AY1*, and *UGT321AP1* were over-transcribed in several resistant CPB populations. To identify TFs that regulate the expression of the three genes, the DNA sequences in the 5′ upstream region of the three genes were cloned and verified, with lengths of −1973/+63 (*CYP9Z140*), −1970/+26 (*CYP9AY1*), and −1799/+173 (*UGT321AP1*). To clarify potential cis-acting element(s) in their upstream regions, recombinant luciferase reporter plasmids containing different fragments of the 5′ regulatory region were constructed. The dual-luciferase reporter assays demonstrated that the promoter constructs (−253/+63 bp), (−1193/+26 bp), and (−578/+173 bp) generated the strongest luciferase activity (*p* < 0.05) compared to other constructs (Figure 1). The binding sites within the key regions of the target gene promoters were predicted through JASPAR (http://jaspar.genereg.net/). The prediction results indicated the presence of multiple binding sites for TFs, including CncC/Maf and FoxO, which co-existed in all three promoters, Abd-B, which co-existed in the promoters of *CYP9Z140* and *CYP9AY1*, and Ptx1, which co-existed in the promoters of *CYP9AY1* and *UGT321AP1*. These binding sites are underlined in the sequence of the three promoters (Figure 2).

### 3.3. Expression Profile of Candidate TFs

**Figure 3:**
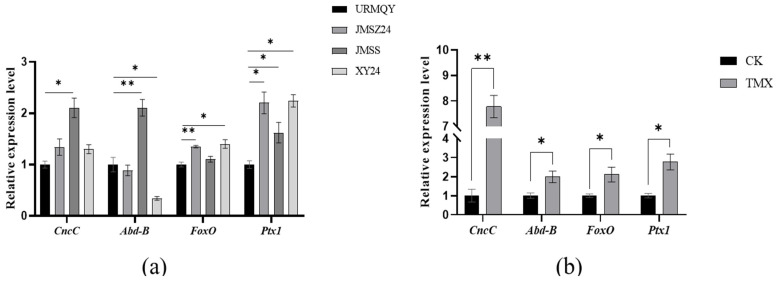
Expression profiles of transcription factors in different field populations (**a**) and following exposure to the LD_50_ of thiamethoxam (**b**) in *L. decemlineata*. Expression levels of the three genes were normalized using *Ef1α* and *RPL4* as internal reference genes. Data are presented as mean ± SE of three biological replicates with three technical replicates. Significant differences are indicated by asterisks (Student’s *t*-test, * *p* < 0.05, ** *p* < 0.01).

**Figure 4:**
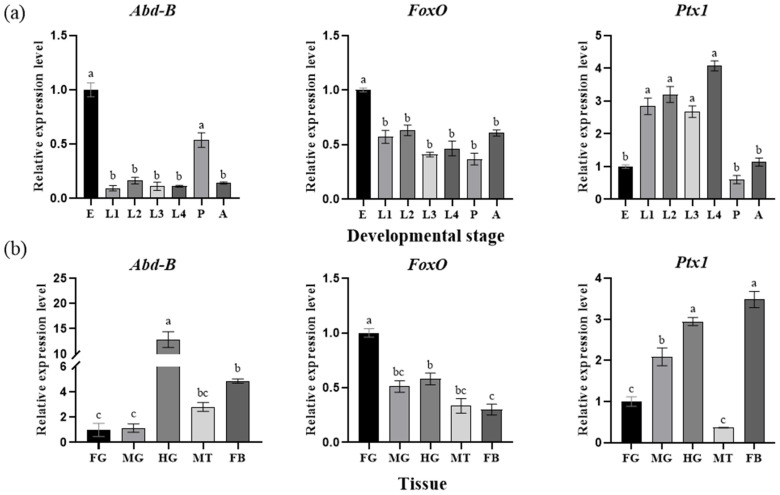
Spatiotemporal expression patterns of transcription factors analyzed by qRT-PCR in *L. decemlineata*. (**a**) Relative expression levels of *Abd-B*, *FoxO*, and *Ptx1* at different developmental stages of CPB. (**b**) Relative expression levels of *Abd-B*, *FoxO*, and *Ptx1* in various tissues of CPB. E: egg; L1-L4: first instar to fourth instar larva; P: pupa; A: adult. FG: foregut; MG: midgut; HG: hindgut; MT: Malpighian tubules; FB: fat body. Data are presented as mean ± SE of three biological replicates with three technical replicates. Bars with different lowercase letters indicate significant differences according to one-way ANOVA followed by Tukey’s multiple comparison test (*p* < 0.05).

First, we cloned and verified the full-length cDNAs of *Abd-B*, *FoxO*, and *Ptx1* (Figures S1–S3), because Sun et al. (2017) [30] had identified *CncC* and its regulatory pathway-related genes in the beetle. To further clarify the stress responses of the four transcription factors under thiamethoxam stress, the expression levels of *CncC*, *Abd-B*, *FoxO*, and *Ptx1* were detected by means of qRT-PCR in populations with different resistance levels and test insects treated with thiamethoxam. The results showed that the mRNA expression of *CncC* and *Abd-B* increased 2.10- and 2.11-fold in the JMSS strain; moreover, *FoxO* in JXYZ24 and XY24 populations and *Ptx1* in JXYZ24, JMSS, and XY24 populations were upregulated significantly by 1.35-, 1.40-, 2.20-, 1.62-, and 2.24-fold compared with the URMQY population, respectively (Figure 3a). In addition, the transcriptional levels of *CncC*, *Abd-B*, *FoxO*, and *Ptx1* increased significantly by 7.7-, 1.98-, 2.11-, and 2.77-fold at 72 h after adults from XY24 were exposed to thiamethoxam LC_50_ (Figure 3b). As the spatiotemporal expression characteristics of *CncC* had been systematically analyzed in the beetle [10], quantitative PCR was further used to analyze the expression patterns of the remaining transcription factors during different developmental stages and in different tissues of *L*. *decemlineata* in this study. The tissue-specific results indicated that the transcriptional levels of *Abd-B*, *FoxO*, and *Ptx1* were highest in the hindgut, foregut, and fat body and hindgut, respectively (Figure 4a). Age-specific expression profiles revealed that the three genes were expressed at all stages of *L*. *decemlineata*. Of them, *Abd-B* was the most highly expressed in the egg and pupa stages, *FoxO* also exhibited the highest expression level in the eggs, and *Ptx1* was highly expressed in the first- to fourth-instar larvae (Figure 4b).

### 3.4. Involvement of Several Candidate TFs in the Upregulation of CYP9Z140, CYP9AY1, and UGT321AP1

**Figure 5:**
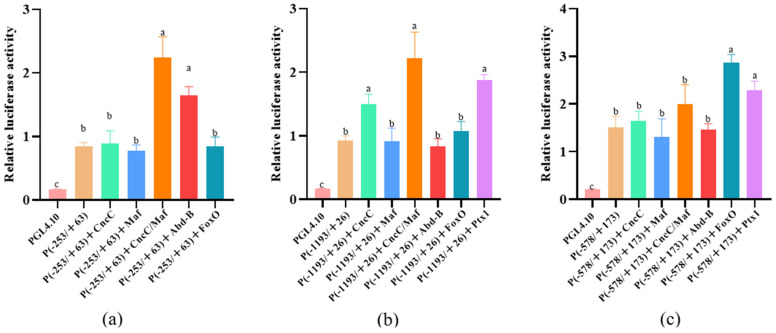
Dual-luciferase reporter analysis of transcription factors regulating promoters of detoxification genes (**a**) *CYP9Z140*, (**b**) *CYP9AY1*, and (**c**) *UGT321AP1* in *L. decemlineata*. The pIEx-4 empty vector was used as a control. Data is presented as mean ± SE of three independent transfections. Bars with different lowercase letters indicate significant differences according to one-way ANOVA followed by Tukey’s multiple comparison test (*p* < 0.05).

To verify whether the above predicted TFs are involved in the transcriptional regulation of *CYP9Z140*, *CYP9AY1*, and *UGT321AP1*, luciferase reporter assays were conducted. The results showed that co-transfection of the CYP9Z140 promoter with CncC/Maf and Abd-B increased luciferase activity by 2.67- and 1.96-fold compared to the pGL4.10 control (Figure 5a). Moreover, CncC, CncC/Maf, and Ptx1 significantly enhanced the promoter activity of *CYP9AY1* by 2.24-, 2.97-, and 5.19-fold (Figure 5b), while co-transfecting the *UGT321AP1* promoter and CncC/Maf, FoxO, and Ptx1 led to 1.44-, 1.60-, and 1.77-fold increases in luciferase activity, respectively (Figure 5c).

**Figure 6:**
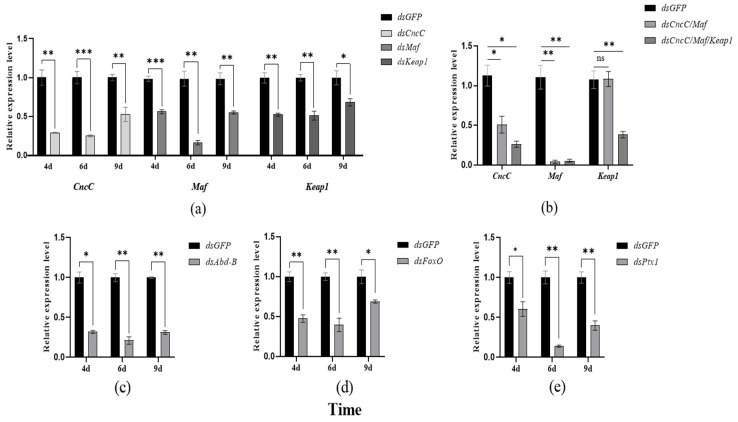
RNA interference efficiency of different transcription factors at various time points in *L. decemlineata*. (**a**,**b**) RNAi efficiency of individual and simultaneous knockdown of *CncC*, *Maf*, and *Keap1*; (**c**–**e**) RNAi efficiency of individual knockdown of *Abd-B*, *FoxO*, and *Ptx1*. Error bars represent the SE from the means of three independent replicates. Significant differences are marked by asterisks (Student’s *t*-test, * *p* < 0.05, ** *p* < 0.01, *** *p* < 0.001). “ns”represents no significant difference between treatment and the control ds*GFP*.

**Figure 7:**
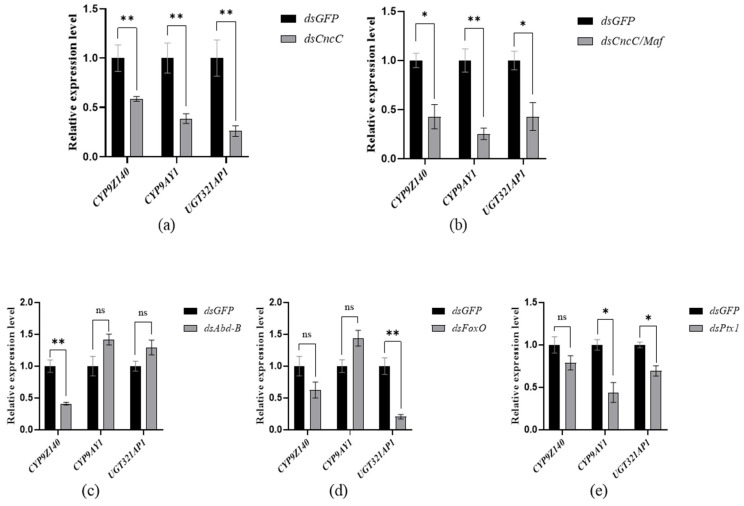
Quantitative PCR analysis of detoxification gene expression after RNA interference targeting different transcription factors in *L. decemlineata*. (**a**) *CncC*; (**b**) simultaneous knockdown of *CncC* and *Maf*; (**c**) *Abd-B*; (**d**) *FoxO*; (**e**) *Ptx1*. The bar labeled in each column indicates the sample mean ± SE of three biological replicates. Asterisks (*) represent significant changes in the mRNA transcript level of each gene in qPCR results (Student’s *t*-test, * *p* < 0.05, ** *p* < 0.01). “ns” represents no significant difference between treatment and the control ds*GFP*.

**Figure 8:**
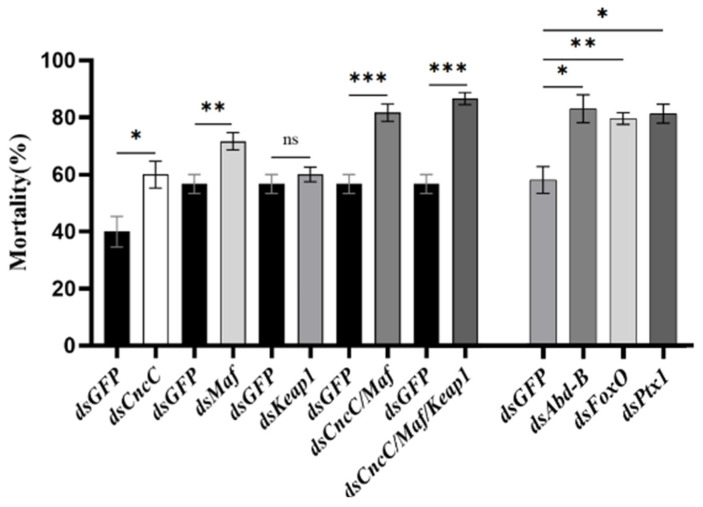
Effects of RNA interference targeting different transcription factors on the susceptibility of *L. decemlineata* to thiamethoxam. Mortality was recorded for adult *L. decemlineata* exposed to thiamethoxam (0.2647 μg/adult) for 72 h after individual and simultaneous RNAi of different transcription factors for 6 d. Data are shown as the mean ± SE of four replicates. Asterisks indicate significant differences (Student’s *t*-test, * *p* < 0.05, ** *p* < 0.01, *** *p* < 0.001). “ns” indicates no significant difference between treatments and ds*GFP*.

The transcription of several TFs was individually or simultaneously suppressed by RNAi, and the mRNA levels of *CYP9Z140*, *CYP9AY1*, and *UGT321AP1* were investigated. The results showed that ingestion of dsCncC, dsMaf, dsKeap, dsAbd-B, dsFoxO, and dsPtx1 for six days resulted in the greatest reduction in the transcription levels of *CncC*, *Maf*, *Keap*, *Abd-B*, *FoxO*, and *Ptx1* by 74.83%, 85.24%, 49.05%, 78.97%, 60.42%, and 86.14%, respectively (Figure 6a,c–e). Simultaneous knockdown of *CncC* and *Maf* also decreased their mRNA levels by 49.12% and 96.09%, respectively, whereas feeding a dsRNA mixture of all CncC pathway genes significantly reduced the expression of *CncC*, *Maf*, and *Keap* by 73.86%, 94.68%, and 61.83%, respectively (Figure 6b). The expression levels of *CYP9Z140*, *CYP9AY1*, and *UGT321AP1* in *CncC* or *CncC*/*Maf* knockdown adults were significantly lower than those in the ds*GFP* control (Figure 7a,b). However, knockdown of *Abd-B* and *FoxO* only resulted in significant downregulation of *CYP9Z140* and *UGT321AP1*, respectively, whereas *Ptx1* silencing simultaneously led to a significant decrease in the expression levels of *CYP9AY1* and *UGT321AP1* (Figure 7c–e). The RNAi results showed that not only individual silencing of *CncC*, *Maf*, *Abd-B*, *FoxO*, and *Ptx1* but also knockdown of *CncC/Maf* and *CncC/Maf/Keap* could result in a significant increase in thiamethoxam susceptibility by 20.00%, 15.00%, 24.21%, 20.88%, 22.54%, 25.00%, and 30.00% compared with ds*GFP*. However, the mortality of adults exhibited no significant difference between ds*Keap* and ds*GFP* (Figure 8).

## 4. Discussion

Metabolic detoxification is an important method by which for insects to counteract insecticides. The related regulation mechanisms of enhanced enzymatic metabolism of insecticides remain incompletely understood. A substantial body of evidence has confirmed that transcription factor-mediated upregulation of P450 genes results in resistance to neonicotinoid insecticides. To date, transcription factors CncC/Maf, CREB, HNF4, and AhR/ARNT have been respectively proven to participate in the regulation of P450 genes mediating imidacloprid susceptibility in insects [10,31,32,33]. CncC/Maf has been identified as a key regulator of *CYP9Z25* and three ABC transporter genes involved in the imidacloprid resistance of *L*. *decemlineata* [8,10]. The transcriptional regulation of detoxification genes is closely associated with insect resistance or tolerance to xenobiotics; however, little is known about *UGTs*. Whether the CncC/Maf-Keap pathway is involved in modulating the expression of *CYP9Z140*, *CYP9AY1*, and *UGT321AP1*, which have been identified as involved in the thiamethoxam resistance of CPB [3], and whether other TFs exist remain unclear.

To elucidate the mechanism underlying the upregulation of *CYP9Z140*, *CYP9AY1*, and *UGT321AP1*, we cloned and verified their promoters and assessed the activity of various truncated fragments of the promoters using a dual-luciferase reporter assay. While all truncated regions exhibited 5′-flanking sequence activity of *CYP9Z140*, *CYP9AY1*, and *UGT321AP1*, the relative activity differed most significantly within the −253 bp to −68 bp, −1193 bp to −819 bp, and −578 bp to −212 bp regions, respectively, suggesting the potential presence of TF-binding sites within these regions. Thereafter, we predicted transcription factors using JASPAR (https://jaspar.genereg.net/), including CncC/Maf, Abd-B, FoxO, and Ptx1, which were predicted to jointly bind to the active promoter regions of the three genes and ranked among the highest scoring candidates. We subsequently cloned the full-length sequences of TFs and predicted and quantified their relative expression via qPCR to preliminarily confirm the link between these transcription factors and the development of thiamethoxam resistance in *L*. *decemlineata* through altered expression of *CYP9Z140*, *CYP9AY1*, and *UGT321AP1*. In this study, the expression levels of *CncC*, *Abd-B*, *FoxO*, and *Ptx1* were found to be significantly higher in the resistant population, XY24, following exposure to thiamethoxam LD_50_ stress compared to the control. The results indicated that the predicted TFs were involved in the resistance of *L*. *decemlineata* to thiamethoxam. The results of the stage expression profile analysis indicate that *Abd-B* and *FoxO* were highly expressed in eggs, whereas *Ptx1* expression was the highest in the first- to fourth-instar larvae. *Abd-B* belongs to the *Bithorax complex* (BX-C) gene cluster of the Hox gene family, which mainly determines the morphological characteristics and appendage development of the insect posterior segment. As the terminal transcription factor of the insulin signaling pathway, FoxO is conserved from *C. elegans* to mammals and plays an important role in a series of physiological, cellular, and pathological processes, including stress resistance, fecundity, lifespan, growth, apoptosis, and innate immunity [17,19,34,35]. The stage expression profile results demonstrate that Abd-B and FoxO play important roles in embryonic development, whereas Ptx1 plays a vital role in the larval development of *L*. *decemlineata*. The tissue-specific expression analysis revealed that *Abd-B* and *FoxO* were highly expressed in the hindgut and foregut, respectively, and *Ptx1* was highly expressed in the hindgut and fat body. The gut and fat body are the main detoxification organs for insecticides in insects, implying that Abd-B, FoxO, and Ptx1 may be involved in insecticide resistance. Our results demonstrated that the spatiotemporal expression peaks of several transcription factors and the regulated detoxification enzyme genes do not completely overlap [3]. Similarly, *LmCncC* from *Locusta migratoria* is highly expressed in the foregut, gastric caecum, and hindgut, whereas LmCncC-regulated *CYP6FD1* and *CYP6FE1* genes related to deltamethrin and imidacloprid resistance are mainly expressed in the Malpighian tubules, fat body, and integument tissues. The authors postulate that CncC potentially participated in the transcriptional regulation of other genes beyond P450s, as the expression peak of CncC is not completely consistent with that of each P450 gene [36].

To further investigate the potential relationship between the predicted transcription factors and the expression of three detoxification enzyme genes, we performed dual-luciferase reporter assays. The assays revealed that CncC/Maf binds to the critical regions of promoters of *CYP9Z140*, *CYP9AY1*, and *UGT321AP1* and significantly enhances their transcriptional activity. In addition, Abd-B and Ptx1 increased the transcriptional activity in the 5′ flanking regions of *CYP9Z140* and *CYP9AY1*, respectively, and Ptx1 and FoxO were able to regulate the transcriptional activity of the *UGT321AP1* promoter. These findings indicate that the specific combinations, including CncC/Maf and Abd-B for *CYP9Z140*, CncC/Maf and Ptx1 for *CYP9AY1*, and CncC/Maf, FoxO, and Ptx1 for *UGT321AP1*, positively regulate the expression of corresponding genes, thereby mediating resistance to thiamethoxam in *L*. *decemlineata*. Similarly, a dual-luciferase reporter assay showed that CncC/Maf promoted the transcription of *GSTe6* from *Spodoptera exigua* [12]. The results of the dual-luciferase reporter assay revealed that FoxO could mainly regulate the expression of *CYP4CE1* with two mutations at positions −648 bp and −2200 bp, thereby mediating nitenpyram resistance in *N. lugens* [20].

Our results demonstrated that dsRNA-mediated knockdown of CncC or CncC/Maf significantly resulted in a concomitant significant reduction in the expression levels of the three genes, *CYP9Z140*, *CYP9AY1*, and *UGT321AP*. The downregulation of *Abd-B* and *FoxO* only led to a reduction in *CYP9Z140* and *UGT321AP* expression, respectively, whereas knockdown of *Ptx1* decreased the expression of both *CYP9AY1* and *UGT321AP*, which is consistent with the results of our dual-luciferase reporter assays. Furthermore, these findings further reveal that CncC is a key transcription factor that regulates the expression of detoxification genes, thereby accelerating phytochemical and insecticide detoxification. However, silencing each candidate TF, excluding *keap*, or a mixture of CncC pathway genes, led to a marked enhancement in the susceptibility of *L*. *decemlineata* to thiamethoxam, implying that CncC/Maf, Abd-B, FoxO, and Ptx1 participate in the thiamethoxam resistance of CPB by regulating the expression of different detoxification genes, respectively. Similar studies demonstrated that CncC has been identified as a transcription factor involved in the regulation of the P450 gene *CYP9Z25* and the ABC transporter genes *ABCH278B*, *ABCH278C*, and *ABCG1041A* responsible for the imidacloprid resistance of CPB by RNAi [8,10]. Our findings add credence to the increasingly recognized notion that the transcription factor CncC is commonly recruited by various insects to cope with pesticide selection by regulating the expression of genes encoding detoxifying enzymes such as P450s and UTGs.

The results of some studies have revealed that FoxO also plays an important role in insecticide resistance. The difference in promoter sequences of *CYP4CE1* between susceptible and resistant populations of *N. lugens* mainly contributed to the different expression levels of *CYP4CE1* regulated by FoxO to develop nitenpyram resistance [20]. *CYP6ER1* in *N. lugens* may be activated by FoxO for imidacloprid resistance and recovered by Akt [37]. It was found that the silencing knockdown of *TcFOXO* and *TcAMPKα* from *T. castaneum* significantly downregulated the expression level of *Tc4EBP* and attenuated its induction by deltamethrin [18].

Additionally, the results of other studies have demonstrated that multiple transcription factors may jointly regulate the expression of genes, thereby mediating the development of resistance. For example, CncC/Maf and AhR/ARNT were found to coordinately regulate the expression of the GST gene *SeGSTe6*, which is involved in chlorpyrifos and cypermethrin resistance in *S. exigua* [12]. In follow-up studies, the authors revealed that CncC/Maf and the POU/homeodomain transcription factor *Nubbin* are associated with the enhanced expression of *CYP321B1*, suggesting that both *trans*- and *cis*-regulatory elements act in combination to modulate the promoter activity of *CYP321B1* in a synergistic manner in *S. exigua* [38]. Deng et al. (2025) [39] also found that AhR/ARNT and CncC/MafK transcriptionally regulated the expression of chemosensory protein genes *CSP2* and *CSP15* involved in imidacloprid resistance in *N. lugens*. Interestingly, the study demonstrated that FoxO not only regulated the mRNA levels of CYP6BQ cluster genes from *T. castaneum*, which are involved in deltamethrin resistance, but also directly regulated the expression of the CncC pathway, a known transcriptional regulator of CYP6BQ genes [19]. Furthermore, to the best of our knowledge, this is the first study in which the involvement of Abd-B and Ptx1 in insecticide tolerance in insects is reported. The previous studies revealed CncC involvement in imidacloprid resistance by regulating genes encoding the P450 enzyme and ABC transporters in CPB [8,10]. Similarly, our research also confirmed the role of CncC/Maf in the thiamethoxam resistance of *L*. *decemlineata*. Additionally, we discovered that three other transcription factors, FoxO, Abd-B, and Ptx1, can jointly regulate the expression of *CYP9Z140*, *CYP9AY1*, and *UGT321AP1* and participate in the development of resistance to thiamethoxam in the beetle.

## 5. Conclusions

In the current study, the dual-luciferase reporter assays and RNAi experiments together confirmed that CncC/Maf is involved in the regulation of *CYP9Z140*, *CYP9AY1*, and *UGT321AP1* expression. This observation strongly demonstrates that the CncC pathway plays an important role in the upregulation of the three genes. In addition, FoxO and Ptx1 regulate *UGT321AP1* expression, whereas Abd-B and Ptx1 could regulate the expression of *CYP9Z140* and *CYP9AY1*, respectively. These results further reveal that multiple transcription factors coordinately regulate the expression of detoxication genes conferring resistance to insecticides in insects. This complex and precise regulatory mechanism involving multiple transcription factors requires further investigation.

## Figures and Tables

**Table 1:** Background information of *L. decemlineata* populations collected from different locations in Xinjiang.

Sampling Date	Population	Sampling Location	Longitude and Latitude
July 2021	URMQY	Yonghe First Brigade, Yongfeng Town, Urumqi, Xinjiang, China	E 87.344529 N 43.60947
June 2024	XY24	Liangfan New Village, Xinyuan County, Yili, Xinjiang, China	E 83.233002 N 43.60947
July 2024	JMSZ24	Louzhangzi Village, Quanzi Street Town, Jimsar County, Changji, Xinjiang, China	E 89.159448 N 43.434803
July 2024	JMSS	Shangjiuhu Village, Quanzi Street Town, Jimsar County, Changji, Xinjiang, China	E 89.1297223 N 43.772049

**Table 2:** Primers used in the study.

Primer Name	Primer Sequence (5′–3′)	Purpose
qCncC-F	CTGAAGATCTCCGTCGTTGAG	qRT-PCR
qCncC-R	CCTTCGCACGATATGGTCTATT	
qMaf-F	TGCAAGCACCTTTGTCACTC	
qMaf-R	CAGGACAACAGGCCAAACAT	
qKeap1-F	CCACGAAATAGAGTGGGAGTG	
qKeap1-R	GGTCCAGTTCCGGATCATAAA	
qAbd-B-F	CTGAGCCTGTTCCGTCTACA	
qAbd-B-R	CCCAACGTTTCTGCTTCGAA	
qFoxO-F	TGGTTCAGAACGTGCCCTAT	
qFoxO-R	GTTTAGCGTCCGGGTTGATC	
qPtx1-F	CTGCTTCAGTCAGGAGTCGA	
qPtx1-R	GCTCATATCGGGGTAGCGAT	
qCYP9Z140-F	ACATGGCCCGAGGAATTGTA	
qCYP9Z140-R	TTTTCAACGGCAAGGACCAC	
qCYP9AY1-F	CATTCGGCATTGGTCCAAGA	
qCYP9AY1-R	CCTTCTGGGCGCATATTGAA	
qUGT321AP1-F	CATCAGGAAATGGCTACCGC	
qUGT321AP1-R	AGACCCACAGCTATGCCTTT	
RPL4-F	AAAGAAACGAGCATTGCCCTTCC	
RPL4-R	TTGTCGCTGACACTGTAGGGTTGA	
Ef1α-F	AAGGTTCCTTCAAGTATGCGTGGG	
Ef1α-R	GCACAATCAGCTTGCGATGTACCA	
ds*CncC*-F	ccatggcggccgcgggaattcCGAAAGGACAGCAACAGC	RNAi
ds*CncC*-R	gctagggtaccaatcgaattcCCTTTTATTACATACTCCCCTG	
ds*Maf*-F	ccatggcggccgcgggaattcGTTGGAGCCGTTGTTGAGTC	
ds*Maf*-R	gctagggtaccaatcgaattcTTCCCATACATCCTTCCATTG	
ds*Keap1*-F	ccatggcggccgcgggaattcGTTGCTGGTATTCACTTA	
ds*Keap1*-R	gctagggtaccaatcgaattcTAGAACTTCTCCCTCTTC	
ds*Abd-B*-F	ccatggcggccgcgggaattcGCAACCTCAATCTCACCGAA	
ds*Abd-B*-R	gctagggtaccaatcgaattcCGGAAAATGCCATAGGTCAC	
ds*FoxO*-F	ccatggcggccgcgggaattcCTTGGACGACCTCAACATCA	
ds*FoxO*-R	gctagggtaccaatcgaattcGAGTACGAAGGTGGAGGTGC	
ds*Ptx1*-F	ccatggcggccgcgggaattcCCTCAGTCAGGAGTCGAACAA	
ds*Ptx1*-R	gctagggtaccaatcgaattcAGGGTGTTGAATTGGGTGC	

**Table 3:** Sensitivity to thiamethoxam of different *L. decemlineata* populations in Xinjiang.

Population	Slope ± SE	LD_50_ (μg/Adult)/(95%FL)	Resistance Ratio (RR)
URMQY	2.1167 ± 0.0528	0.0311(0.0238–0.0408)	1.00
JMSZ24	2.5857 ± 0.006	0.0498 (0.0386–0.0643)	1.60
JMSS	2.7853 ± 0.0217	0.2079 (0.1695–0.25521)	6.69
XY24	2.3515 ± 0.0375	0.2647 (0.2005–0.3493)	8.51

Note: RR = The LD_50_ of the test population/the LD_50_ of the relatively sensitive population.

## Data Availability

All data presented in this study are available in this article.

## Supplementary Materials

The following supporting information can be downloaded at: https://www.mdpi.com/article/10.3390/insects17050525/s1. Figure S1: Bioinformatics analysis of Abd-B in *L. decemlineata*; Figure S2: Bioinformatics analysis of FoxO in *L. decemlineata*; Figure S3: Bioinformatics analysis of Ptx1 in *L. decemlineata*.

## Appendix A

**Table A1:** Primers used for the dual-luciferase reporter assay.

Primer Name	Forward Primer (5′–3′)	Reverse Primer (5′–3′)
CYP9Z140-Fa (−68/+63)	tacctgagctcgctagcctcgagTGCATCCGACTGTTTTCATTT	aacagtaccggattgccaagcttCACTGTCTGAAACCTGACGCTA
CYP9Z140-Fb (−253/+63)	tacctgagctcgctagcctcgagAACCCACATCTCAAAAAGGAAA	
CYP9Z140-Fc (−830/+63)	tacctgagctcgctagcctcgagTAATCAAAAGAAAGCGTGCGAA	
CYP9Z140-Fd (−957/+63)	tacctgagctcgctagcctcgagCTGGAAATCAAATAAGCCGTT	
CYP9Z140-Ff (−1651/+63)	tacctgagctcgctagcctcgagGCAAGAGGCAGGCACTTAGAA	
CYP9Z140-Fg (−1973/+63)	tacctgagctcgctagcctcgagCACCAAGTTCAACACTTCCTCA	
CYP9AY1-Fa (−145/+26)	tacctgagctcgctagcctcgagCAGTTGGCTACTCGGGAAAAT	aacagtaccggattgccaagcttATCGATATGGGTATTCCACCGA
CYP9AY1-Fb (−819/+26)	tacctgagctcgctagcctcgagCTCAACAAACCCAATCTAACAGC	
CYP9AY1-Fe (−1193/+26)	tacctgagctcgctagcctcgagTCAGGATGTAACTCCCCTGTG	
CYP9AY1-Ff (−1677/+26)	tacctgagctcgctagcctcgagTACCTGATCCTTGAGATAGACGA	
CYP9AY1-Fg (−970/+26)	tacctgagctcgctagcctcgagTTCAGGAGTCGATGAGGAGGA	
UGT321AP1-Fa (−212/+173)	tacctgagctcgctagcctcgagCTCAACTTCAGCCTCCGCTAC	aacagtaccggattgccaagcttCAGCCTCCTTGAATGACGGTA
UGT321AP1-Fb (−578/+173)	tacctgagctcgctagcctcgagGCCTGAACCACTGTGCATGA	
UGT321AP1-Fe (−949/+173)	tacctgagctcgctagcctcgagCCTACAGGTGTATTCGGTCCAT	
UGT321AP1-Ff (−1232/+173)	tacctgagctcgctagcctcgagTAGACCTACCTACCCTTTGAGAA	
UGT321AP1-Fg (−1497/+173)	tacctgagctcgctagcctcgagGACTGTCGGAAGACAGGGTGT	
UGT321AP1-Fh (−1799/+173)	tacctgagctcgctagcctcgagTTGTCTCTGGCTAGGAAGGGTA	
CncC	cgcggatcccaattggcagatctGCATGAATTAAAAGTGCGCTAG	agcttcgaaccggtaccgtcgacTTATCAAGGAAAATTGGTGAGTC
Maf	cgcggatcccaattggcagatctTCACATCAAAAAGGTGAACC	agcttcgaaccggtaccgtcgacAGGCCAAACATTTCTGTAGG
Abd-B	cgcggatcccaattggcagatctGCCAGTTTCCATCAAGCCAC	agcttcgaaccggtaccgtcgacGCTACCCGGACAAAGTAGCA
FoxO	cgcggatcccaattggcagatctTGCTGACTTGTGGATCTGTG	agcttcgaaccggtaccgtcgacAATCCTACGTTAATGCACCC
Ptx1	cgcggatcccaattggcagatctCGGAGACTTTACAGCATCAA	agcttcgaaccggtaccgtcgacTTCCTTACCTCGTCAATCGT

**Table A2:** Primers used for the reverse transcriptase polymerase chain reaction (RT-PCR).

Gene	Forward Primer (5′–3′)	Reverse Primer (5′–3′)
Abd-B	CCAGTTTCCATCAAGCCAC	GCTACCCGGACAAAGTAGCA
FoxO	TGCTGACTTGTGGATCTGTG	AATCCTACGTTAATGCACCC
Ptx1	CGGAGACTTTACAGCATCAA	TCCTTACCTCGTCAATCGT
