# Supplementary material for: Identification of Transcription Factors Regulating Detoxification Genes CYP9Z140, CYP9AY1, and UGT321AP1 Involved in Thiamethoxam Resistance in Leptinotarsa decemlineata
Source: Insects. 2026 May 20;17(5):525. doi: 10.3390/insects17050525 (PMC13207126; doi:10.3390/insects17050525)
Supplement: Supplementary file 1 [file insects-17-00525-s001.zip › insects-4306490-supplementary.pdf]

(a)

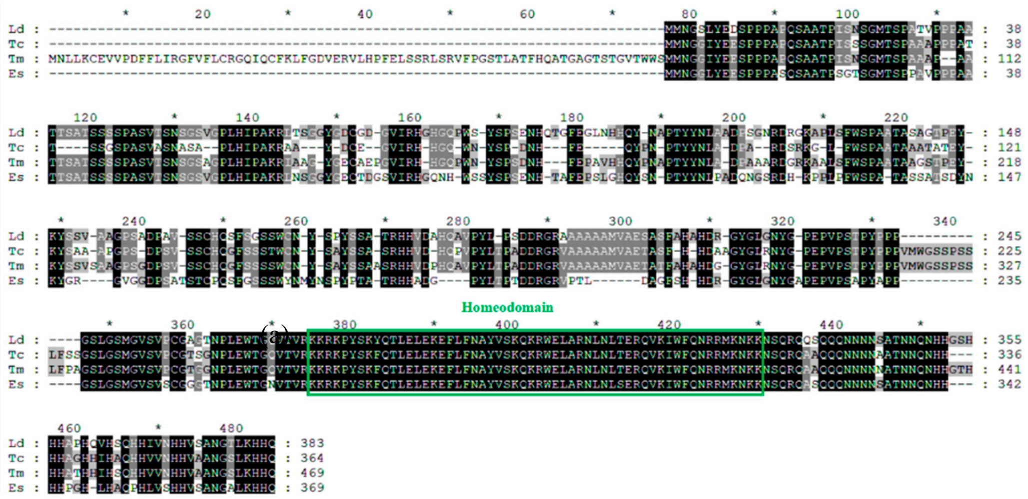

(b)

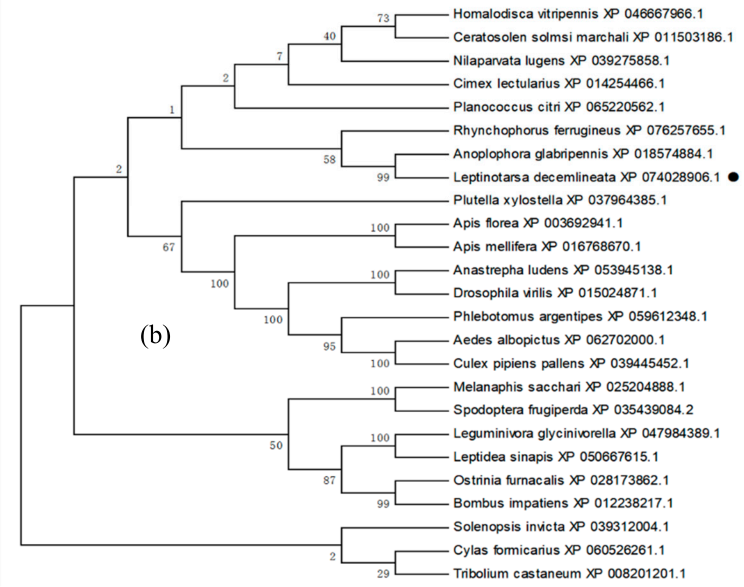

Figure S1. Bioinformatics analysis of Abd-B in *L. decemlineata*. (a) Amino acid sequence alignment of the *Abd-B* gene. The homodomain is highlighted by a green box. Ld: *Leptinotarsa decemlineata*; Tc: *Tribolium castaneum*; Tm: *Tenebrio molitor*; Es: *Euwallacea similis*. (b) Phylogenetic tree of Abd-B proteins from *L. decemlineata* and other insects. Bootstrap values (1000 replicates) are indicated beside the branches, and GenBank accession numbers are shown in parentheses. The black dot indicates Abd-B in *L. decemlineata*.

(a)

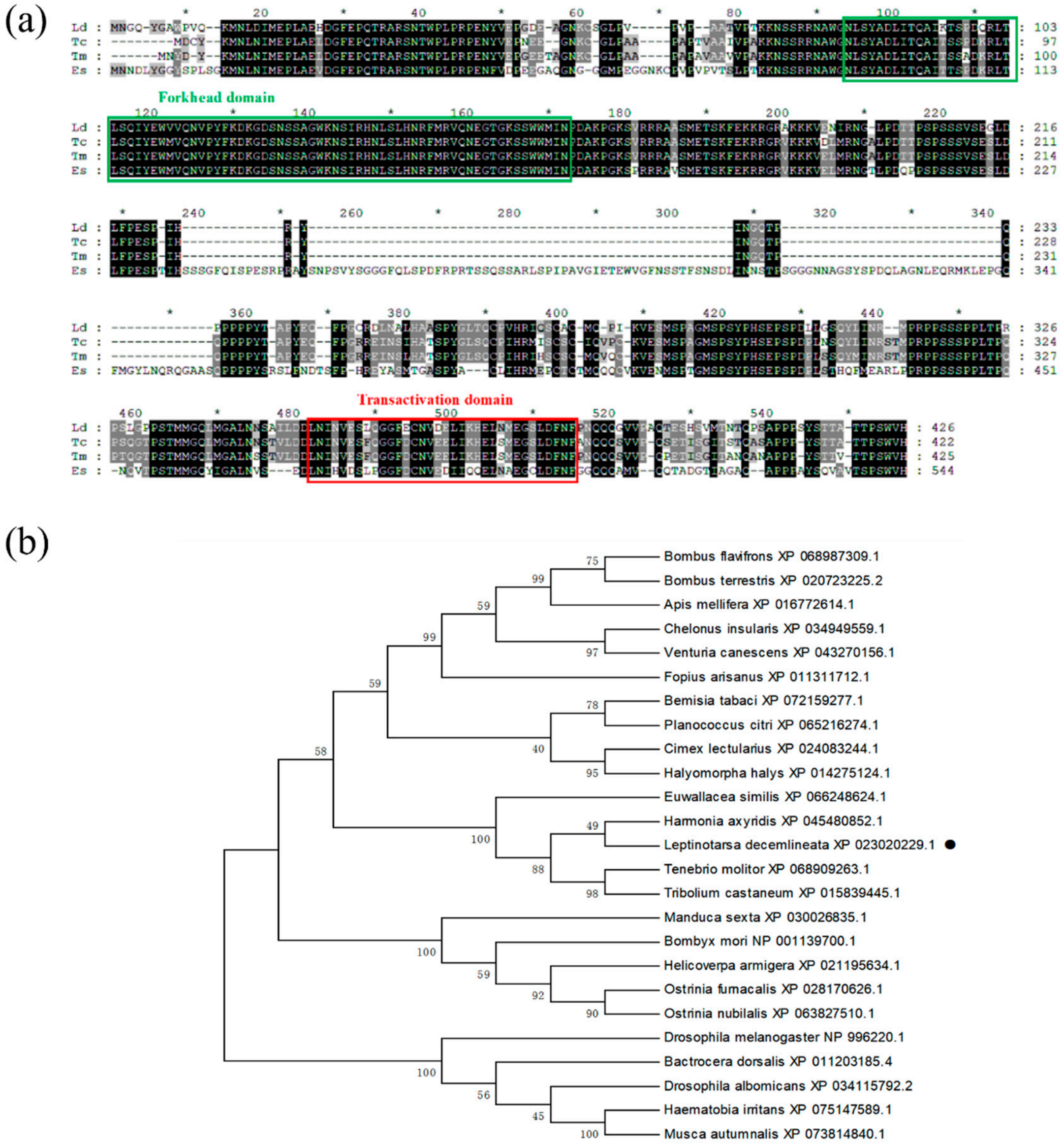

Figure S2. Bioinformatics analysis of FoxO in *L. decemlineata*. (a) Amino acid sequence alignment of the *FoxO* gene. The Forkhead domain is highlighted by a green box, and the transactivation domain is marked by a red box. Ld: *Leptinotarsa decemlineata*; Tc: *Tribolium castaneum*; Tm: *Tenebrio molitor*; Es: *Euwallacea similis*. (b) Phylogenetic tree of FoxO proteins from *L. decemlineata* and other insects. Bootstrap values (1000 replicates) are indicated beside the branches, and GenBank accession numbers are shown in parentheses. The black dot indicates FoxO in *L. decemlineata*.

(a)

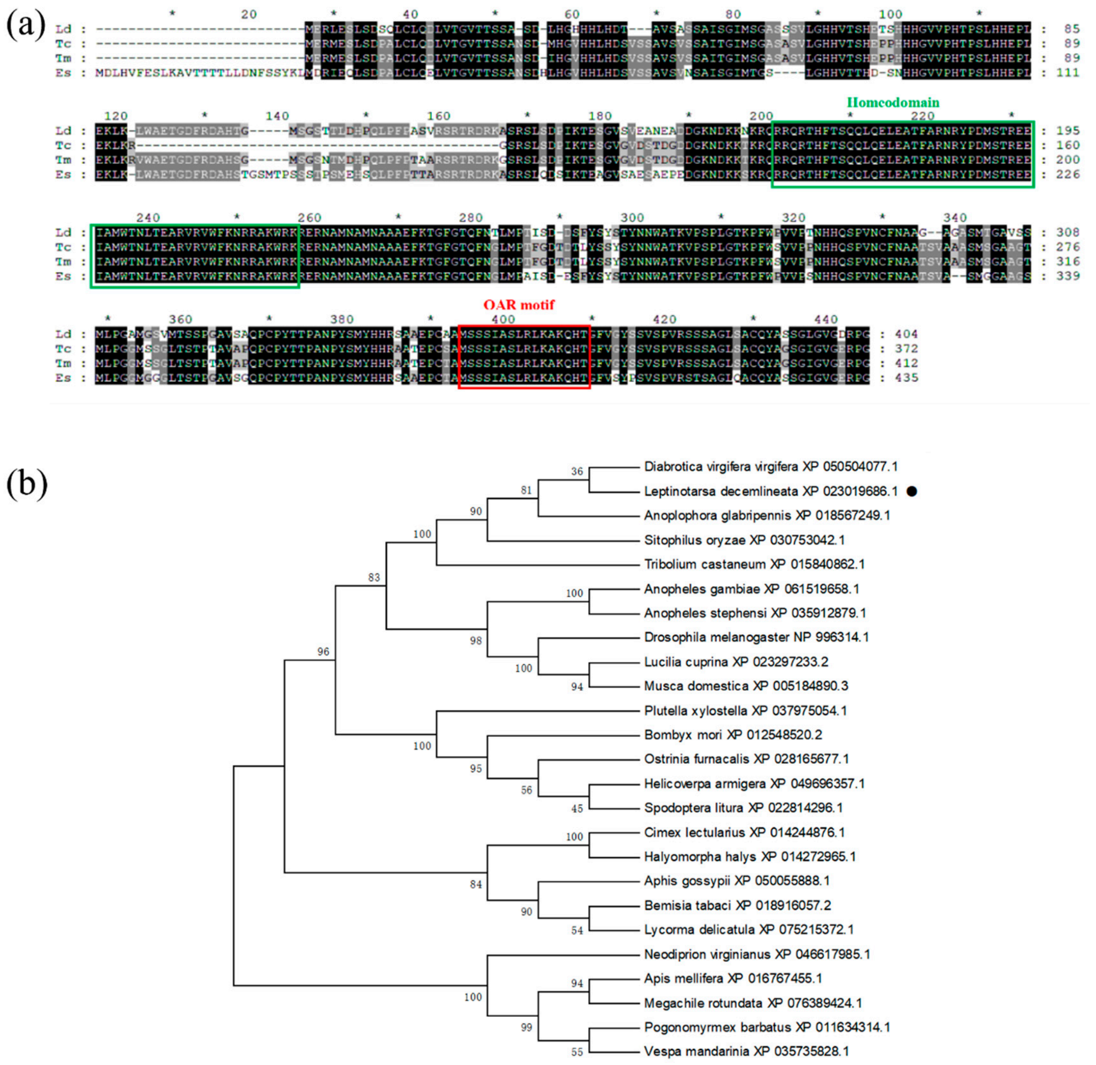

Figure S3. Bioinformatics analysis of Ptx1in *L. decemlineata*. (a) Amino acid sequence alignment of the *Ptx1* gene. The homeodomain of *Ptx1* is highlighted by a green box, and the OAR modification site is marked by a red box. Ld: *Leptinotarsa decemlineata*; Tc: *Tribolium castaneum*; Tm: *Tenebrio molitor*; Es: *Euwallacea similis*. (b) Phylogenetic tree of Ptx1 proteins from *L. decemlineata* and other insects. Bootstrap values (1000 replicates) are indicated beside the branches, and GenBank accession numbers are shown in parentheses. The black dot indicates Ptx1 in *L. decemlineata*.
